# Synthesis of Hierarchical SnO_2_ Nanowire–TiO_2_ Nanorod Brushes Anchored to Commercially Available FTO-coated Glass Substrates

**DOI:** 10.1007/s40820-017-0136-6

**Published:** 2017-02-21

**Authors:** Derek R. Miller, Sheikh A. Akbar, Pat A. Morris

**Affiliations:** Department of Materials Science and Engineering, The Ohio State University, Columbus, OH 43212 USA

**Keywords:** SnO_2_, Nanowire, Fluorine-doped SnO_2_ (FTO), Vapor–liquid–solid (VLS), TiO_2_, Nanorod brush

## Abstract

Growth of single-crystal SnO_2_ nanowires using a fluorine-doped SnO_2_ (FTO) thin film as both the source and substrate is demonstrated for the first time at relatively low temperature (580 °C) which preserves the integrity of the underlying glass support and improves scalability to devices. Furthermore, a microwave hydrothermal process is shown to grow TiO_2_ nanorods on these nanowires to create a hierarchical nanoheterostructure that will lead to efficient photogenerated charge carrier separation and rapid transport of electrons to the substrate. This process simplifies nanowire growth by using commercially available and widely used FTO substrates without the need for an additional upstream Sn source and can be used as a high surface area host structure to many other hierarchical structures.
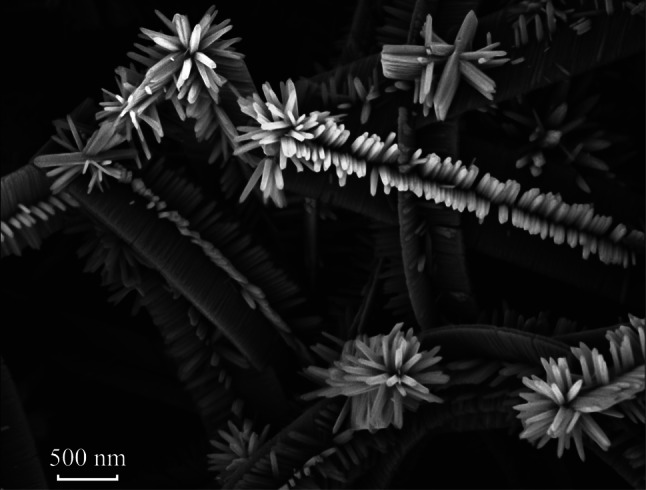

## Highlights

- Single-crystal SnO_2_ nanowires can be grown from polycrystalline thin films of fluorine-doped SnO_2_ (FTO) on glass substrates at a reduced temperature of 580 °C without the need for an upstream source powder.
- A novel hierarchical nanobrush-like structure was designed to maximize the surface area of the photoactive material while aiding more efficient photogenerated charge carrier separation and extraction through the nanowire core for photocatalysis and dye-sensitized solar cells. 
- The SnO_2_ nanowires were used as an anchored 3D host to create a novel hierarchical nanobrush-like structure, and this immobilized structure was designed to maximize the surface area of the photoactive material while aiding more efficient photogenerated charge carrier separation and extraction through the nanowire core for photocatalysis and dye-sensitized solar cells.

## Introduction

Stannic oxide (SnO_2_) has been greatly studied in many fields for its electronic, catalytic, and optical properties [1–3]. It is often employed as a transparent conducting oxide (TCO) by doping with indium (ITO) or fluorine. These transparent substrates are heavily used in optoelectronic applications as transparent conductors such as thin film LEDs and dye-sensitized solar cells (DSSC) [4–6]. In DSSC applications, it is imperative that nanomaterials can effectively separate photogenerated electron–hole pairs and that the electrons can be harvested into the external circuit from the nanomaterials before recombination or trapping in defect states [7]. To this end, high-quality single-crystal nanowires or nanorods are often employed as the active material [8, 9]. However, nanowires deposited as a slurry are randomly oriented and do not necessarily lead to the TCO layer or the external circuit. TiO_2_ nanorods have been grown, deposited, or attached to FTO substrates in a number of clever ways including double-sided brush-like flakes via hydrothermal reaction [10, 11] and around carbon fibers via microwave hydrothermal reaction [12]. Carney et al. [13] originally showed that Au-tipped SnO_2_ nanowires could be grown directly from pressed and sintered 95% SnO_2_–5% CoO pellets with a sputtered Au thin film through a vapor–liquid–solid (VLS) mechanism at 700–800 °C. This method greatly simplifies nanowire growth compared to many other techniques that utilize an upstream source and downstream substrate [14, 15], often needing independently controlled temperature zones to induce condensation [16]. The present work shows that a modification of this method can grow single-crystal SnO_2_ nanowires from polycrystalline thin films of fluorine-doped SnO_2_ (FTO) on glass substrates at a reduced temperature of 580 °C. These nanowires are then used as an anchored 3D host to create a novel hierarchical nanobrush-like structure using hydrothermally grown TiO_2_ nanorods. This immobilized structure is designed to maximize the surface area of the photoactive material while aiding more efficient photogenerated charge carrier separation and extraction for photocatalysis and dye-sensitized solar cells. Photogenerated electrons should move into the single-crystal SnO_2_ nanowire core due to a larger work function, leaving the separated hole to react with the solution or dye at the surface. The high-quality core nanowire can then help shuttle the electrons directly to the thin film on which it is anchored and grown, where it can then be collected to pass through an external circuit. Similar morphologies of nanoheterostructures have been synthesized with different combinations of materials previously and led to unique or enhanced properties [17–19]. This is a promising and simple process for creating nanoheterostructures on commercially available and widely used FTO substrates that may also lead to enhanced properties in photocatalysis, DSSCs, and gas sensing.

## Experimental Section

**Fig. 1:**
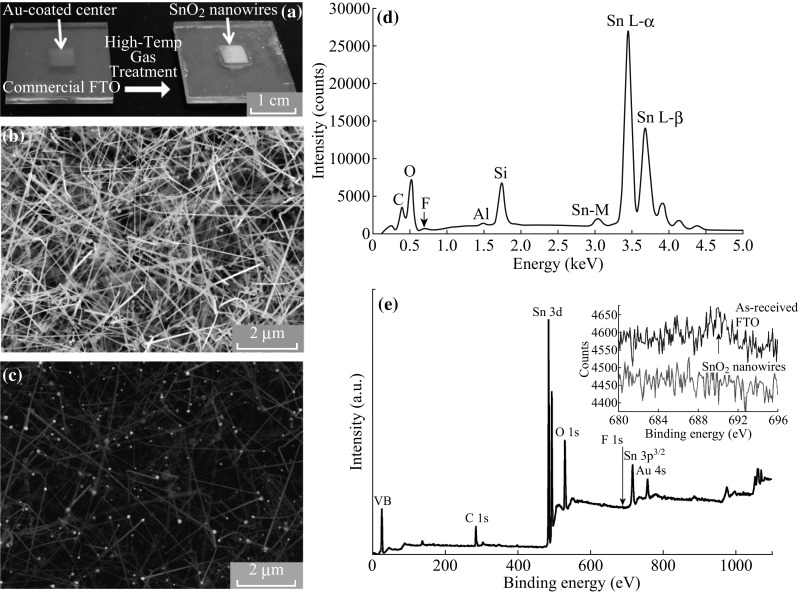
**a** FTO-coated glass slides showing growth from the slide Au-coated while masked (*left*) to the nanowire-covered center (*right*). **b** Secondary electron image of surface covered in SnO_2_ nanowires. **c** Backscattered electron image of the same area, showing gold nanoparticle tips. **d** EDS spectrum of as-received FTO with the small fluorine bump denoted by an* arrow*. **e** XPS survey spectrum of as-received FTO showing the fluorine region of the as-received and SnO_2_ nanowires* inset*

SnO_2_ nanowires were grown from a polycrystalline FTO-coated glass substrate by a high-temperature gold-catalyzed VLS mechanism. The FTO glass slides (TEC 7, Hartford Glass Company) were square shape with 2.25 cm sides and 2.2 mm thick, as shown in Fig. 1a. The polycrystalline FTO coating was measured to be 600 nm thick. The slides were cleaned by sonication for 5 min each in acetone, ethanol, and H_2_O and then dried in an oven at 140 °C. The slides were sputter-coated with a ~15-nm Au coating using a Pelco Model 3 Sputter Coater. A Kapton™ tape mask was placed over the surface during sputtering to restrict gold-catalyzed growth to a pre-defined region in the center. The substrate was loaded horizontally into a quartz tube with 2.54 cm inner diameter with no other precursors present. For optimal growth, the quartz tube was heated to 580 at 5 °C min^−1^ under zero flow conditions. When the growth temperature was reached, 750 sccm 5% H_2_/N_2_ was flowed through a room-temperature upstream water bubbler to humidify the gas and was held for 8–12 h to facilitate growth of the nanowires. The gas flow was then shut off and the furnace cooled at 2 °C min^−1^. The reported conditions were suitable to grow gold-tipped rutile SnO_2_ nanowires approximately 20–100 μm long and 40–80 nm in diameter as shown in Fig. 1b.

The preparation of FTO precursor solution for a sacrificial coating followed that as described previously [4]. SnCl_4_ ·5H_2_O (99.99%), NH_4_F (99.99%), and ethanol (99.5%) were purchased from Sigma–Aldrich. Microwave hydrothermal growth of TiO_2_ nanorods was carried out using a CEM Discover SP microwave digestion system. TEM samples were prepared by sonicating the nanowires or powders into a dispersion in methanol and dropping the dispersion onto 3-mm copper lacey carbon grids, followed by drying at 140 °C. TEM electron diffraction was performed by an FEI/Phillips CM200T. X-ray diffraction (XRD) was performed using a Rigaku Smart Lab with Cu-*K*α radiation (*λ* = 1.5408 Å).

## Results and Discussion

The growth of the nanowires is attributed to the well-known VLS mechanism as evidenced by the Au-tipped nanowires. The main difference from previous studies is that the source of the Sn and O is from the substrate itself, rather than from an upstream source powder. The source/substrate FTO is a relatively stable oxide, so the 5% H_2_/N_2_ is needed to reduce the oxide to induce vaporization as SnO or Sn metal, while the humidified flow provides the necessary oxygen to re-oxidize the material pushed out of the Au nanoparticles into nanowires. This atmosphere is likely the key to allowing a lower growth temperature than other studies.

**Fig. 2:**
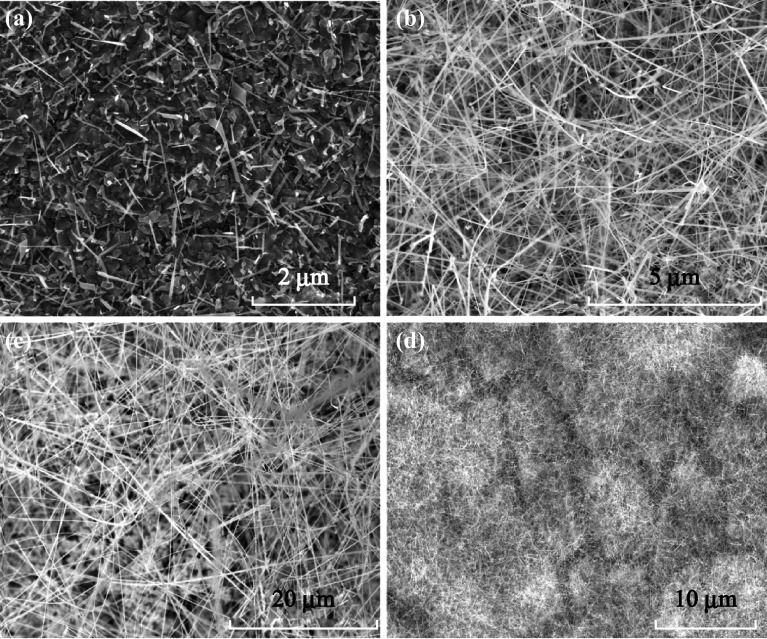
SnO_2_ nanowires grown on FTO at **a** 520 °C, **b** 620 °C, **c** 670 °C with no catalyst, and **d** from additional solution of FTO spin-coated onto the original thin film grown at 570 °C

A wide range of growth conditions were initially investigated before settling on the optimal conditions as described in “Experimental section.” The main challenge was to achieve dense growth of long nanowires while minimally affecting the glass substrate and FTO thin film. The resistance of the FTO film was measured with point probes at opposite corners of the FTO film to assess the damage to the film’s electronic properties. The as-received FTO slides had a measured resistance of 30 Ω. After a 12-h growth at 520 °C and gas flow as described previously, producing very few nanowires (Fig. 2a), the resistance increased to 60 Ω. At the lowest successful nanowire growth temperature (580 °C, Fig. 1), the film resistance further increased to 490 Ω. These measurements were all on substrates whose nanowire growth was restricted to the center of the slide, like those shown in Fig. 1a. Growth temperatures above 675 °C completely destroyed the integrity of the FTO film and slightly softened the glass, but grew a very dense coating of nanowires **(**Fig. 2c). Nanowire growth without the catalyst layer was only possible above 620 °C.

The FTO thin film was measured at roughly 600 nm thick by tracking the ratio of Sn (FTO) to Si (glass) in an energy-dispersive X-ray spectroscopy (EDX) line scan of the substrate cross section and also directly imaged using the electron backscatter detector. EDX analysis in Fig. 1d of the as-received FTO thin film detected only a small bump in signal where a fluorine elemental peak is expected, but not significant enough to be assigned by the program. X-ray photoelectron spectroscopy (XPS) shown in Fig. 1e also detected only a small increase in signal at 687 eV. This is compared to that of the nanowires, which shows no increase, inset in Fig. 1e. These methods are barely sensitive enough to detect any presence of fluorine in the as-received FTO, so it is unknown if any of the original fluorine doping was incorporated into the nanowires.

To reduce the degradation of the FTO thin film, an additional solution-based FTO layer was spin-coated over the original layer to act as a sacrificial source for growth. Sacrificial coatings were added using approximately 60 µL of solution via pipette onto the substrate while on a spin coater at 500 rpm. Although the catalyst layer was still necessary, this additional layer assisted growth of longer nanowires and allowed adequate growth at slightly lower temperatures of 560–570 °C, as shown in Fig. 2d. However, even if the sacrificial layer protected the underlying thin film, the resistance of the top layer was quite high at 3 kΩ (560 °C) and 20 kΩ (570 °C), likely due to the poor quality of the spin-coating that leaves many microcracks after drying and annealing. Careful modification of the thickness of the deposited coating and growth conditions may eventually achieve growth solely from the sacrificial layer without affecting the high-quality transparent thin film. This coating was also applied to a bare Si wafer, and after gold sputtering, nanowire growth was achieved at the same conditions as on the FTO slide. This demonstrates that a simple solution-based coating can be used to grow SnO_2_ nanowires on nearly any substrate that is stable above 580 °C under reducing conditions without the need for an upstream precursor or a reaction chamber with multiple independent temperature-controlled zones.

TiO_2_ nanorods were precipitated onto the SnO_2_ nanowires using a microwave hydrothermal method similar to that described previously [9]. However, the previous study employed traditional hydrothermal growth in a PTFE autoclave over 1–24 h and grew nanorods directly on a blank FTO slide. This was first replicated in the microwave hydrothermal chamber, and then a slide with pre-grown SnO_2_ nanowires was used as the growth substrate. The SnO_2_ nanowire-covered FTO substrates were placed upright into a quartz vial reaction chamber containing a solution of 5 mL H_2_O, 5 mL HCl, and 0.5 mL titanium (IV) butoxide. The inner diameter of the quartz tube was 2.54 cm, which allowed two FTO slides to stand upright back-to-back with no additional support. This prevented TiO_2_ deposition on the back of the slides, allowed a small magnetic stir bar to freely spin in the tapered bottom of the vial, and prevented settling of large TiO_2_ particles on the surface of the slides. A typical growth experiment used 150 W of intermittent microwave power to hold the reaction chamber temperature at 150 °C for 5 min with the resulting pressure holding between 150 and 250 psi. Reaction times greater than 5 min did not increase the coverage of nanorods on the nanowires, suggesting the nucleation and growth is complete after that period. Nanorods did not sufficiently grow at temperatures below 150 °C or pressure below 150 psi, but higher pressure and temperature did not change the morphology. The previously cited study [9] conducted extensive analysis of varying reaction parameters and growth mechanisms of the TiO_2_ nanorods, so the most promising formula and reaction conditions were chosen for this study, but achieved instead via microwave heating. The reader is referred to this paper [9] for more details on the nucleation and growth process of the nanorods.

**Fig. 3:**
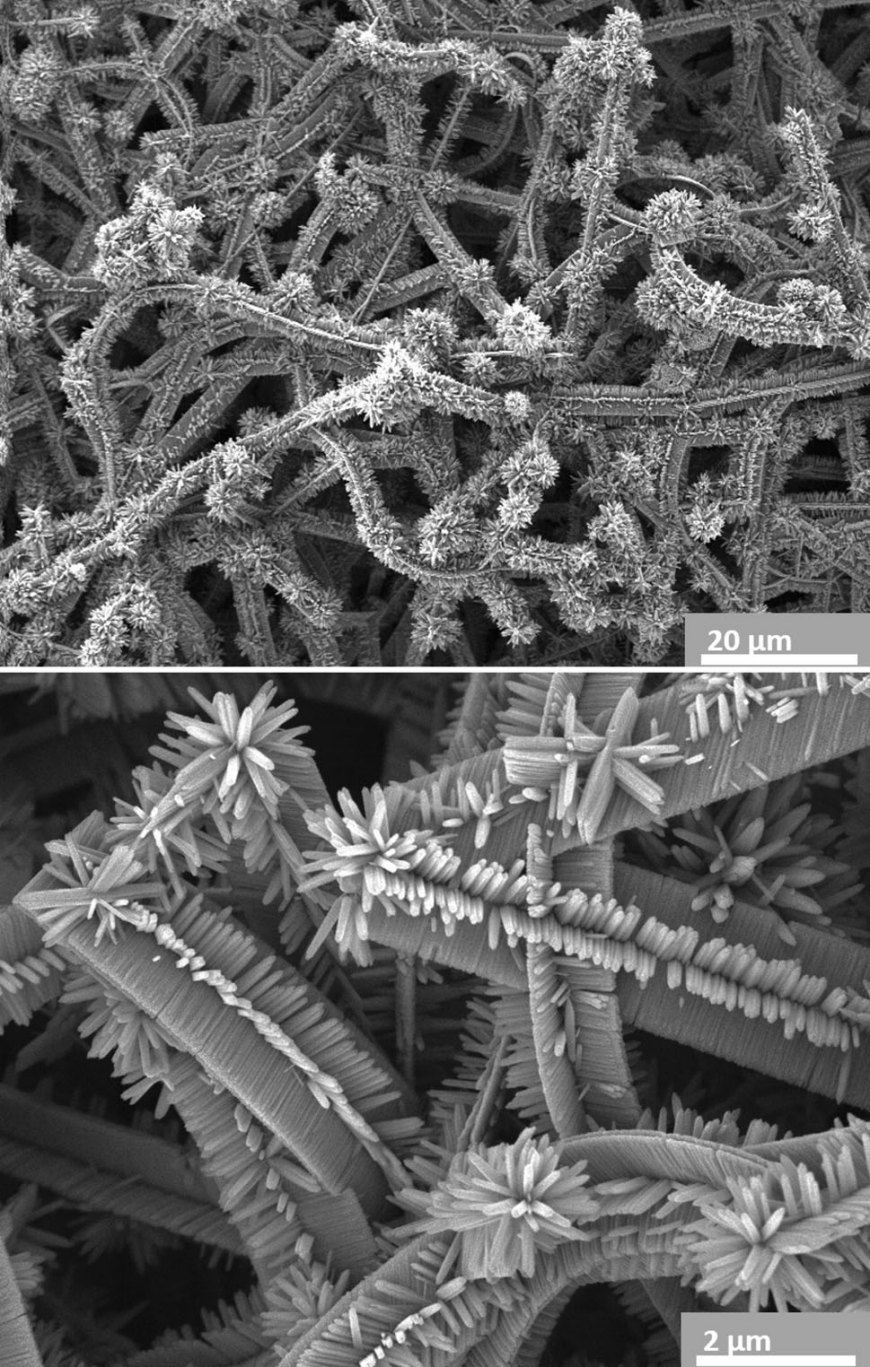
Hierarchical structure consisting of TiO_2_ nanorods grown radially from SnO_2_ nanowires anchored on FTO thin film-coated glass slides

After the reaction, the slides were promptly removed from solution and rinsed with ethanol and DI water. Figure 3 shows the SEM images of the intricate hierarchical structures created by this multi-step process. The TiO_2_ nanorods grow outward in a quasi-sixfold radial pattern from the core SnO_2_ nanowires. Analysis of many regions of several growth iterations shows that the nanorods first nucleate on the nanowires and grow exactly opposite (180°) to each other in dense rows on either side of the core nanowire, and additional nanorods then grow in a “V” shape on either side of this flat surface in rows that are less densely packed than the first. The degree of nanorod precipitation on each nanowire depends on the precursor concentration, growth time, and height of the area in solution. Regions closer to the bottom of the reaction chamber tended to have less growth.

**Fig. 4:**
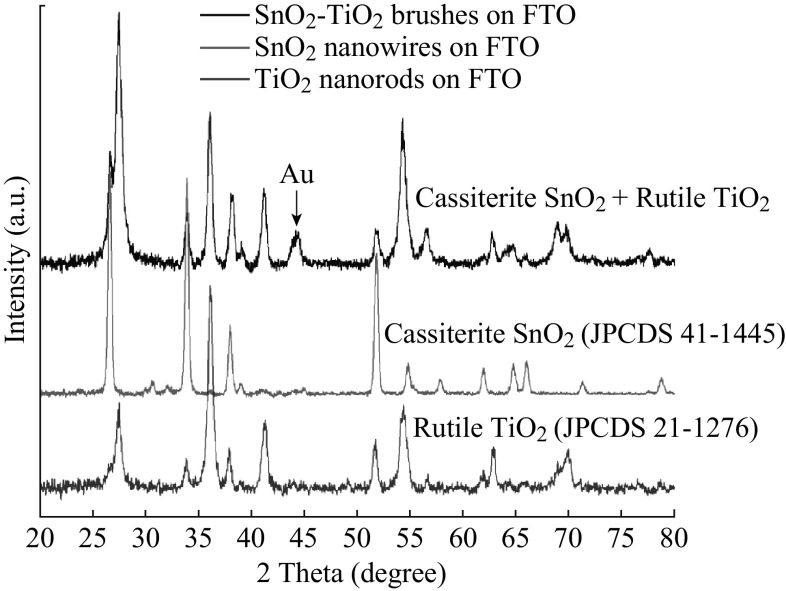
XRD patterns from: (*bottom*) TiO_2_ nanorod film grown hydrothermally directly on FTO; (*middle*) plain SnO_2_ nanowires grown from FTO via VLS; (*top*) SnO_2_ nanowire–TiO_2_ nanorod brushes synthesized via two-step VLS and hydrothermal process

XRD was carried out on the plain SnO_2_ nanowires, the TiO_2_ nanorods grown directly on the FTO thin film, and the nanoheterostructure brushes, shown in Fig. 4. The plain SnO_2_ nanowires clearly show the expected cassiterite (rutile) phase (JPCDS 41–1445). The TiO_2_ nanorods show the tetragonal rutile phase (JPCDS 21–1276). The heterostructure clearly shows the combination of peaks from both of these phases, confirming that it neither undergoes any unexpected phase transformations. An extra peak from the Au nanoparticle tips is also visible at 44.6° (200).

**Fig. 5:**
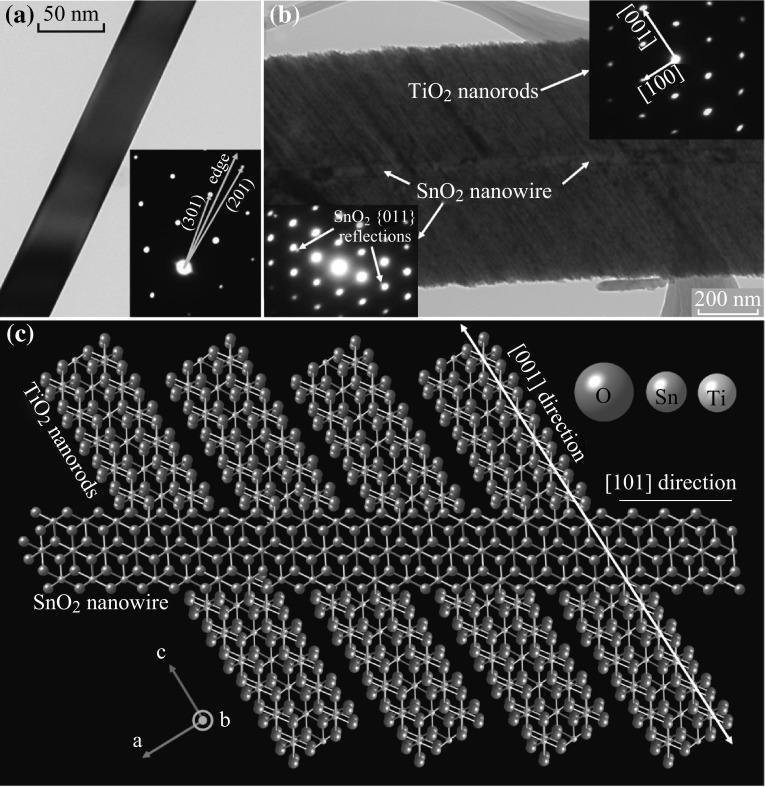
**a** TEM image of pristine SnO_2_ nanowire with inset SAED pattern showing a growth direction pointing toward the (502) reflection, whose plane normal corresponds to a [101] axis. **b** TEM image of SnO_2_–TiO_2_ brush with one plane of TiO_2_ nanorods growing at 33° from perpendicular to the nanowire axis.* Inset top right* shows the [001] growth direction of the TiO_2_ nanorods and* inset bottom left* shows the faint reflections when an area including both the nanowires and nanorods is selected, showing that they have the same orientation. **c** An atomic model showing the epitaxial relationship of the angled nanorods growing from the SnO_2_ nanowire looking down the [010] zone axis

Selected area electron diffraction (SAED) was used to analyze the phases and crystallographic relationship between the core SnO_2_ nanowires and the outwardly radiating TiO_2_ nanorods. Analysis of ten pristine SnO_2_ nanowires showed a common <101> growth direction and rutile (cassiterite) structure (Fig. 5a), the same as reported previously using an upstream source and a silicon substrate [14, 20–22]. Diffraction studies on ten hierarchical brushes showed a common <001> growth axis on rutile TiO_2_ nanorods nucleated on the nanowires (Fig. 5b) as well as those in self-nucleated clusters, which agrees with similar studies [9, 11, 12]. The initial flat plane of nanorods grows outward from the nanowires in two fashions: directly perpendicular to the nanowire growth axis and at 33° from perpendicular. SAED of the core nanowire was largely dominated by the diffraction of the surrounding nanorods, but faint reflections were found on the inner edge of the first-order TiO_2_ {011} spots that were not present when the selected area did not include the core nanowire. Since both the TiO_2_ and SnO_2_ phases here are rutile and TiO_2_ has slightly smaller lattice parameters, the SnO_2_ {011} reflection would be expected 6% closer to the direct beam than the {011} TiO_2_ reflection. Careful measurements of the pattern in Fig. 5b (inset bottom left) show that these faint reflections are indeed 6% closer and measure to the correct *d*-spacing of 0.378 Å^−1^. The {020} reflections are predicted to be only 2.9% closer and are likely obscured by the intensity of the signal from the TiO_2_. Crystal structure calculations show that the (101) plane is approximately 33° from the (002) plane. Interestingly, the angled nanorods are at the same crystallographic orientation as the core nanowires, as shown in the atomic model in Fig. 5c. The [010] zone axis was the most commonly found in the plain nanowires and was almost always found for nanorods attached to the nanowires. The growth direction of the SnO_2_ nanowires yields {101} and {010} surface facets. If the [010] zone axis was nearly always found with brushes whose nanorods lie in the plane of the TEM screen, then that leads to the conclusion that the nanorods are primarily attaching to the {101} facets of the nanowire. This model suggests that the nanorods use their {101} anchoring facet to nucleate and primarily grow outward epitaxially from the {101} SnO_2_ surface facet. Roughly half of the nanorods grow perfectly perpendicular to the nanowire and so are not able to share an epitaxial relationship. These nanorods also held the [010] zone axis while lying flat in the plane of the TEM screen, suggesting that they do not rotate about their [001] axis in this attachment angle. The nanorods seem to completely surround the core nanowire, so it is possible that the nanorods simply nucleate and grow epitaxially to their neighbors directly outward from their nucleation point. Many brushes have additional nanorods growing out-of-plane in a less controlled fashion, but typically forming a “V” shape, as shown in Fig. 3. The angle between the original flat plane of nanorods and the “V”-shaped nanorods is approximately 57°, and the acute angle of the “V” measured to roughly 66°, suggesting that the additional “V”-shaped nanorods grow by attaching their (101) facets. A similar study found that the TiO_2_ nanorods often contained (101) capping facets at their tip [10]. The formation of the “V”-shaped secondary nanowires was not as strong when the primary plane grew in the slanted orientation as shown in Fig. 5. Nevertheless, strong orientation relationships exist in both cases suggesting well-formed, stable interfaces that should be conducive to charge carrier movement. Future studies should focus on developing these promising nanoheterostructures into devices to evaluate their full potential.

## Conclusions

This study has demonstrated a simple process for growing high-quality SnO_2_ nanowires anchored directly to a FTO thin film. Additional solution-deposited FTO coatings offer a versatile method to grow SnO_2_ nanowires from any substrate that can withstand the processing conditions. The novel hierarchical nanobrushes consisting of SnO_2_ nanowires and hydrothermally grown TiO_2_ nanorods are promising for future applications of photocatalysis, water splitting, and dye-sensitized solar cells (DSSCs). The remaining challenge is to fabricate such a device without affecting the quality or properties of the thin film or substrate. This could be overcome by fabricating a thicker FTO film that is not as susceptible to degradation with nanowire growth. The underlying glass substrate could also be substituted with sapphire or quartz to allow growth at higher temperatures without softening of the slide. Future studies will explore the properties of this nanostructure and its implications in fields such as catalysis, photocatalysis, and gas sensing.
